# Intestinal Ecology Changes in Diarrheic Père David’s Deer Revealed by Gut Microbiota and Fecal Metabolites Analysis

**DOI:** 10.3390/ani12233366

**Published:** 2022-11-30

**Authors:** Junai Zhen, Xueli Yuan, Liping Tao, Huidan Zhang, Yijun Ren, Shengbin Xie, Libo Wang, Hua Shen, Yuqing Chen

**Affiliations:** 1 Jiangsu Province Key Laboratory for Molecular and Medical Biotechnology, Life Sciences College, Nanjing Normal University, Nanjing 210000, China; 2 Dafeng National Nature Reserve, Yancheng 224100, China

**Keywords:** microbial diversity, lipids metabolism, acylcarnitines, bile acids

## Abstract

**Simple Summary:**

Père David’s deer *(Elaphurus davidianus*) are classified as extinct in the wild from the International Union for Conservation of Nature Red List. In 2021, there were more than 6000 individuals in Dafeng Reserve, China. With the increasing of population size for Père David’s deer in Dafeng Reserve, it is urgently needed to conduct disease research in order to protect this animal better. Diarrhea is one of the most common diseases affecting the health of Père David’s deer. However, little is known about how the intestinal ecology changes in these diarrheic animals. The aim of this study is to reveal the changes of intestinal microbiome and metabolic pathways in diarrheic Père David’s deer based on gut microbiome and metabolic pathways. Using 16S rRNA gene sequencing and ultra-high performance liquid chromatography combined with tandem mass spectrometry, the gut microbiota and fecal metabolites were analyzed in five diarrheic Père David’s deer. Results demonstrated the distinct changes in the diversity and composition of gut microbiota, as well as great changes in numerous fecal metabolic profiles in diarrheic Père David’s deer. The integrated pathway analysis revealed serious disturbances in several metabolic pathways, such as lipid, bile acid, cofactor and vitamin metabolism. These data provided important gut ecology information for diarrheic Père David’s deer, which may facilitate improved diagnostic and treatment strategies for sick animals in the future.

**Abstract:**

Diarrhea is one of the most common diseases affecting the health of Père David’s deer (*Elaphurus davidianus*). It is believed that an imbalanced intestinal ecology contributes to the etiology of the condition. However, little is known about how the intestinal ecology changes in these diarrheic animals. In this study, 16S rRNA gene sequencing and ultra-high performance liquid chromatography combined with tandem mass spectrometry (UPLC-MS/MS) were used to investigate the gut microbiota and fecal metabolites in five Père David’s deer with diarrhea. The results showed that when compared with healthy individuals, considerable changes in the gut microbiome were observed in diarrheic animals, including a significant reduction in microbial diversity and gut microbiota composition alterations. Furthermore, the profiles of numerous fecal metabolites were altered in diarrheic individuals, showing large-scale metabolite dysregulation. Among metabolites, acylcarnitines, lysophosphatidylcholine, bile acids, and oxidized lipids were elevated significantly. Constantly, several metabolic pathways were significantly altered. Interestingly, predicted metabolic pathways based on 16S rRNA gene sequence and differential metabolite analysis showed that lipid metabolism, cofactor, and vitamin metabolism were altered in sick animals, indicating microbiota-host crosstalk in these deer. When combined, the results provide the first comprehensive description of an intestinal microbiome and metabolic imbalance in diarrheic Père David’s deer, which advances our understanding and potential future treatment of diarrheic animals.

## 1. Introduction

Père David’s deer (*Elaphurus davidianus*) are listed as extinct in the wild according to the International Union for the Conservation of Nature (IUCN) Red List. After experiencing the extinction of wild populations in China in around 1900, 77 captive Père David’s deer overseas were reintroduced to China from 1985 to 1987, and then several reserves were established [1]. After 40 years’ effort, the population size of Père David’s deer exceeded 8000 in China in 2020 [1,2]. In Dafeng Reserve, there were 6119 individuals of the semi-free population and wild population in 2021 (http://www.chinamlw.org/, accessed on 19 June 2021). Although the Père David’s deer population has increased, their genetic diversity is relatively low because of the small population size of founder individuals [3]. Therefore, it is urgently needed to strengthen the management of the population health of Père David’s deer in order to protect this animal better. Some disease research has been conducted in Père David’s deer, revealing that certain diseases, especially infectious diseases, can fatally impact the Père David’s deer population [4]. To date, several pathogens, including parasites (such as *Toxoplasma gondii*, *Cryptosporidium* spp, *Eimeria coccidiosis*, and *Fasciola hepatica*) [5,6,7,8], pathogenic bacteria (such as *Clostridium perfringens*) [9], and virus (such as bovine viral diarrhea virus-like strains) [10], have been detected in serum, feces, or tissue of Père David’s deer. These pathogens are potential threats to the health of Père David’s deer. A study showed the digestive tract diseases to be the primary factor for the deaths of Père David’s deer in captivity [11]. Therefore, in addition to possible pathogen identification, several studies were conducted to reveal the gut microbiome of Père David’s deer under different conditions, such as different diets [2], captive versus wild [12], or different areas [11]. However, there are currently no detailed studies on the intestinal ecology in Père David’s deer under disease conditions, including digestive tract diseases.

Diarrhea is usually defined as the passage of loose stools, and it is the common symptom in Père David’s deer. A captive study in Dafeng Reserve reported that the diarrheal incidence in newborn Père David’s deer (6 days old), after artificial weaning, was approximately 94.74%, and diarrheic mortality was approximately 9.72% [13]. However, no further study was conducted to provide deeply understanding for the diarrhea in this Reserve. Usually, diarrhea is a complex disease that can be induced by multiple factors, including infectious and non-infectious causes. Infection with a harmful microorganism or pathogen is one such cause, while digestive disorders and some diseases are examples of non-infectious causes [14]. Animal gastrointestinal (GI) tracts contain large, complex microbial communities essential for host health maintenance [15]. In healthy organism guts, microbiota create protective barriers against infectious agents; however, microbiota dysbiosis, as well as disturbances in the metabolic harmony of microbial communities, is implicated in several diseases in human, such as inflammatory bowel diseases (IBD) and diarrhea [16,17]. Traditional diarrhea research has focused on individual pathogens, and traditional efforts to unravel diarrheic episodes in the deer family have focused on individual diarrhea pathogens, with bacteria, viruses, and parasites reported in white-tailed deer (*Odocoileus virginianus*), musk deer (*Moschus berezovskii*), and red deer (*Cervus elaphus*) species [18,19,20]. Meantime, intestinal microbial communities are often severely imbalanced in diarrheic hosts, regardless of the diarrhea cause; even gut microbiota dysbiosis may be causative for diarrhea [21,22]. The changes of gut microbiota were reported in diarrheic Baer’s pochards (*Aythya baeri*) [23], musk deer (*Moschus berezovskii)* [19] and giraffes (*Giraffa camelopardalis reticulata*) [24]. However, to date, the gut microbiota condition in diarrheic Père David’s deer is still unknown.

Due to the convenience, noninvasiveness, and sufficient biomass for analysis, feces are the major source of samples for intestinal microecology studies, especially in wild animals [2,25]. Previously, it was shown that fecal metabolomic profiles are generated by functional activities in both host cells and gut microbiota, thereby reflecting gut microbiota composition and activity to a large extent [26]. Gut microbiota and associated metabolites interact with host metabolic processes and influence host health [27]. Recently, a gut microbiota and fecal metabolomics combination study was conducted for human IBD-associated diarrhea, and provided the most comprehensive analysis of host and microbial activities in IBD [28]. However, little is known about the intestinal metabolic changes in Père David’s deer with diarrhea, which hamper the evaluation of the nutritional status and the development of treatment strategies for diarrheic individuals.

In this study, we examined the gut microbiota and fecal metabolites in five captive diarrheic Père David’s deer using 16S rRNA gene sequencing and ultra-high performance liquid chromatography combined with tandem mass spectrometry (UPLC-MS/MS). Results demonstrated the dramatic changes in gut microbiota in diarrheic individuals. More importantly, the changes of intestinal metabolism in diarrheic Père David’s were revealed for the first time, which providing important data for guiding treatment of Père David’s with diarrhea.

## 2. Materials and Methods

### 2.1. Sample Collection

In October 2020, five Père David’s deer (between 6 and 8 months old) in the captive area of Dafeng Reserve developed diarrhea symptoms at the same time, diagnosed by professional veterinarians. Because there are no healthy deer of the same age in this captive area, five healthy deer of nearly 2 years old were selected as the controls. Same food (silage together with grass) and water source were provided for all the deer in our study. Fecal samples were collected on the third day after diarrhea onset. One day prior to sample acquisition, healthy and diarrheic deer were placed in separate pens to prevent sample contamination. Diarrheic deer did not receive any drug treatments (antibiotics and/or anti-inflammatory drugs) prior to sample collection. To ensure sample freshness, feces were immediately collected after defecation. Briefly, the core of the fecal material was collected in a sterile tube with the sterile spoon to avoid lateral exposure to the air. Then samples were placed on dry ice and transported to the laboratory within 24 h. The samples were divided into 200 mg portions and kept at −80 °C until further use. In addition, 15 feces samples from non-diarrhea individuals were also collected from semi-wide and wild areas in Dafeng Reserve. Relative moisture content was determined as follows: 250 mg fresh feces was dried at 60 °C of for 12 h, then the dry feces were weighed. The fecal relative moisture content was calculated as: (fecal wet weight–fecal dry weight)/fecal wet weight. Then these 15 samples were divided into 3 groups based on feces texture and relative moisture content. The research complies with the agreement made by the China Wildlife Conservation Association and the legal requirements of China.

### 2.2. 16S rRNA Gene Amplification and Sequencing 

The total DNA was extracted from fecal (200 mg) using a Magnetic Soil and Stool DNA Kit (TIANGEN, Beijing China). 2% agarose gels were used for electrophoretic DNA visualization, and a NanoDrop 2000 UV-vis spectrophotometer (Thermo Scientific, Waltham, MA, USA) for DNA concentrations. Then, DNA was used as a polymerase chain reaction (PCR) template to amplify the V3-V4 region of 16S rRNA gene using 338F (5′-ACTCCTACGGGAGGCAGCA3′) and 806R (5’ -GGACTACHVGGGTWTCTAAT-3’) [29]. PCR products (250 bp–300 bp) were purified quantified, and used to construct a sequencing library using a TruSeq^®^ DNA PCR-Free Sample Preparation Kit (Illumina Inc., San Diego, CA, USA). After the library was qualified, sequencing was performed on a NovaSeq6000 (Illumina) according to manufacturer’s instructions.

### 2.3. Bioinformatics Analysis 

Raw sequencing reads were merged using FLASH (v1.2.7) and quality control was performed using QIIME software (V1.9.1) to generate high-quality reads by filtering short (<150 base-pairs), homopolymeric, chimeric, and ambiguous sequences using default settings. In total, 776,818 high-quality reads were obtained, with a mean of 81,377 reads/diarrheic sample, and a mean of 73,986 reads/healthy sample. Using the QIIME pipeline, operational taxonomic units (OTUs) were assigned based on at least a 97% sequence similarity level. OTU sequences were annotated to species using the Mothur method, and the Small Subunit rRNA (SSUrRNA) database SILVA138 [30] was used to perform species annotation (0.8~1 threshold was set), and taxonomic information was obtained and analyzed at each classification level (phylum, family, and genus). Gut microbial α-diversity (i.e., Chao1 and Shannon indices), weighted UniFrac distances, and Unweighted Pair-group Method with Arithmetic Means (UPGMA) tree construction were calculated and constructed, respectively, using QIIME software. Simultaneously, a rarefaction curve for each sample was generated to assess sequencing depth. Principal component analysis (PCA) was performed in the R package. The Tax4Fun R package was used to generate functional annotations, and t-test differential analyses were performed on functionally annotated genes.

### 2.4. UPLC-MS/MS Analysis

Approximately 50 mg of sample was homogenized in 500 μL ice-cold methanol/water (70%, *v*/*v*) plus an internal standard (Sigma-Aldrich, Saint Louis, MO, USA). Samples were then vortexed, sonicated, and centrifuged at 12,000 rpm for 10 min at 4 °C. The supernatant was filtered through a 0.2 µm filter and then 150 μL sample was used for UPLC-MS/MS (MS/MS, QTRAP^®^, AB Sciex, Framingham, MA, USA) analysis as previously [2]. Linear ion trap (LIT) and triple quadrupole (QQQ) scans were acquired on a QQQ-linear ion trap MS, equipped with an electrospray ionization (ESI). In total, 599 metabolites were identified and annotated according to the MetWare database (http://www.metware.cn/, accessed on 31 March 2022).

### 2.5. Metabolomics Data Analysis

A supervised multivariate method, orthogonal partial-least square discriminant analysis (OPLS-DA) was performed using R packages to maximize metabolome differences between diarrheic and healthy samples. The relative importance of each metabolite in the PLS-DA model was checked using the variable importance in projection (VIP) parameter. VIP values, extracted from OPLS-DA data, were generated in the R package MetaboAnalystR. Differential metabolites between groups were determined using VIP ≥ 1 and absolute Log_2_FC (fold change) ≥1 values. Unsupervised PCA (principal component analysis) was performed using the statistics function prcomp in R (www.r-project.org, accessed on 8 April 2022) to identify features showing maximum variation between samples. Differential metabolites were annotated and sorted using the Kyoto Encyclopedia of Genes and Genomes (KEGG) database (https://www.genome.jp/kegg/, accessed on 8 April 2022). Metabolic pathway analyses were performed using KEGG pathway enrichment based on differential metabolites. Significantly enriched pathways were identified using a hypergeometric test *p*-value for a given list of metabolites. Spearman’s correlation coefficients of differential metabolites and microbiomes were calculated in R software to generate heat maps and network diagrams.

### 2.6. Conventional PCR and Quantitative Real Time PCR 

*Bacteroides*-specific primers, F2 primer (5’-CAACCCTTGCCGTTAGTTGC-3’) and R2 primer (5′-TGTAAGGGCCGTGCTGATTT-3′) based on *Bacteroides* 16S rRNA gene sequence were designed for conventional PCR and quantitative real-time PCR (qRT-PCR). Conditions for the conventional PCR were 95 °C for 3 min, then 30 cycles of 95 °C for 15 s, 50 °C for 15 s and 72 °C for 30 s, followed by a final amplification of 72 °C for 5 min, and cooled down to 4 °C finally. PCR products were visualized in 1% agarose gels. SYBR Green PCR Kit was used for qPCR. The reaction was performed using a StepOne Plus Real-Time PCR system. Briefly, 10 μL reactions (5 μL of SYBR Green qPCR Master Mix, 1 μL of DNA, 3.6 μL ddH_2_O, 0.2 μL of forward and reverse primers) was subjected to one cycle of 95 °C for 5 min, then 40 cycles of 95 °C for 10 s, 60 °C for 30 s, and followed by 95 °C for 15 s, 60 °C for 60 s and 95 °C for 15 s. Relative gene levels were calculated based on the 2^−ΔΔCt^ method. 

### 2.7. Statistical Analysis

Statistical analysis of data was performed using R (v3.0.3) and GraphPad Prism (version 7.0c). A *p* < 0.05 value was considered statistically significant, and the values were expressed as the mean ± SEM from 4-6 independent experiments.

## 3. Results

### 3.1. Gut Microbiome Differences and Diversity in Diarrheic Père David’s Deer

**Figure 1:**
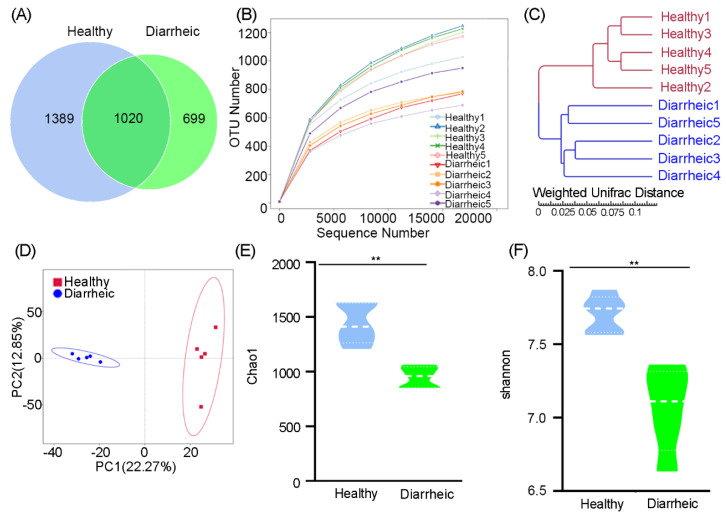
Gut bacterial OTU distribution, feasibility, and diversity analyses. (**A**) Venn diagram showing OTU composition. (**B**) Rarefaction curve showing sequencing quality based on OTU abundance. (**C**) Clustering based on weighted UPGMA analysis. (**D**) PCA score plots of OTUs. α-Diversity comparisons based on Chao1 (**E**) and Shannon indices (**F**). Data were presented as the mean ± SEM (*n* = 5 animals/group). ** *p* < 0.01.

In total, 3108 OTUs were identified at the 97% sequence similarity level. The number of sequences identified at the genus level ranged from 549–876 (mean = 649 sequences) in diarrheic samples, whereas in healthy samples, the range was 500–572 (mean = 537). An OTU Venn diagram showed that 1020 OTUs belonged to healthy samples and 699 OTUs to diarrheic samples. These data reflected the lower, unique OTUs in diarrheic samples (Figure 1A). A rarefaction curve showed that with increasing sequences, the curve tended to be flat, indicating that samples were fully sequenced, the sequencing depth was basically covered, and fewer undetected species were detected, thereby indicating reliable sequencing (Figure 1B). UPGMA analysis, based on the weighted UniFrac distance, indicated that diarrheic and healthy samples were clearly clustered into their own groups (Figure 1C). PCA showed that the five diarrheic samples were separated from the five healthy samples using principal coordinate 1, and tended to form two apparent clustering arrangements (Figure 1D). Therefore, more similar fecal microbiota were present in the five diarrheic individuals than in the five healthy individuals, indicating distinct gut microbiome differences between groups.

Microbial community richness, indicated by the Chao1 index, showed significantly lower levels in diarrheic samples relative to healthy samples (960.84 ± 107.98 vs. 1412.25 ± 222.16, *p* = 0.0009, Figure 1E). Community diversity, estimated by the Shannon index, was also significantly lower in diarrheic samples than in healthy samples (6.92 ± 0.34 vs. 7.74 ± 0.18, *p* = 0.0018, Figure 1F). Therefore, gut microbiota richness and diversity were significantly lower in diarrheic samples than healthy samples.

### 3.2. Altered Microbiota Composition in Diarrheic Père David’s deer

**Figure 2:**
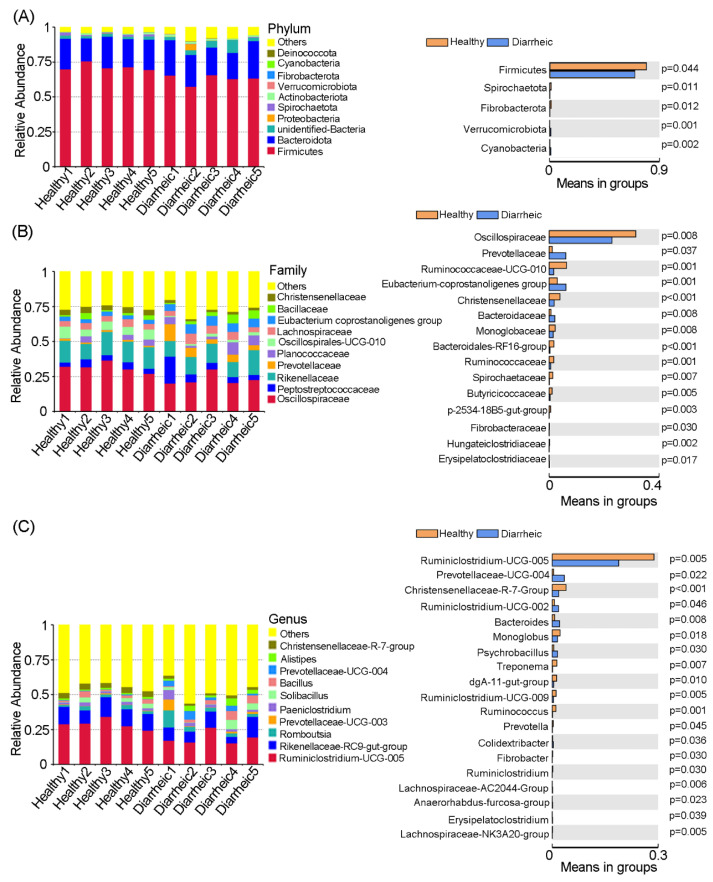
Gut microbiota comparisons between groups. Bacterial composition and comparisons were conducted at phyla (**A**), family (**B**), and genus (**C**) levels based on relative abundance. Data were presented as the mean ± SEM (*n* = 5 animals/group).

We identified 14 bacterial phyla in all feces samples. Among these, Firmicutes and Bacteroidetes were core phyla in samples and accounted for >90% abundance. When compared with the healthy group, the relative abundance of Firmicutes (*p* = 0.044), Spirochaetota (*p* = 0.011), and Fibrobacterota (*p* = 0.012) was significantly lower in the diarrheic group, while the relative abundance of Verrucomicrobiota (*p* = 0.001) and Cyanobacteriain (*p* = 0.002) was significantly higher (Figure 2A). 

At the family level, significant differences in several families were observed between groups (Figure 2B). When compared with the healthy group, the relative abundance of Oscillospiraceae (*p* = 0.008), Ruminococcaceae-UCG-010 (*p* = 0.001), Christensenellaceae (*p* < 0.001), Monoglobaceae (*p* = 0.008), Bacteroidales-RF16-group (*p* < 0.001), Ruminococcaceae (*p* = 0.001), Spirochaetaceae (*p* = 0.007), Butyricicocccaceae (*p* = 0.005), and Fibrobacteracease (*p* = 0.030) was significantly lower in the diarrheic group, while Prevotellaceae (*p* = 0.037), Eubacterium-coprostanoligenes-group (*p* = 0.001), and Bacteroidaceae (*p* = 0.008) abundance was significantly higher.

At the genus level, the relative abundance of 19 bacterial genera was significantly different between groups. The relative abundance of *Ruminiclostridium-UCG-005* (*p* = 0.005), *Christensenellaceae-R-7-group* (*p* < 0.001), *Monoglobus* (*p* = 0.018), *Treponema* (*p* = 0.007), *dgA-11-gut-group* (*p* = 0.010), *Ruminiclostridium-UCG-009* (*p* = 0.005), *Ruminococcus* (*p* = 0.001), *Prevotella* (*p* = 0.045), and *Ruminiclostridium* (*p* = 0.030) was significantly lower in diarrheic samples, while the relative abundance of *Prevotellaceae-UCG-004* (*p* = 0.022), *Ruminiclostridium-UCG-002* (*p* = 0.046), *Bacteroides* (*p* = 0.008), *Psychrobacillus* (*p* = 0.030), and *Colidextribacter* (*p* = 0.036) was significantly higher (Figure 2C). Therefore, distinct gut microbiota compositional changes were observed in diarrheic Père David’s deer.

### 3.3. Altered Fecal in Diarrheic Père David’s deer

**Figure 3:**
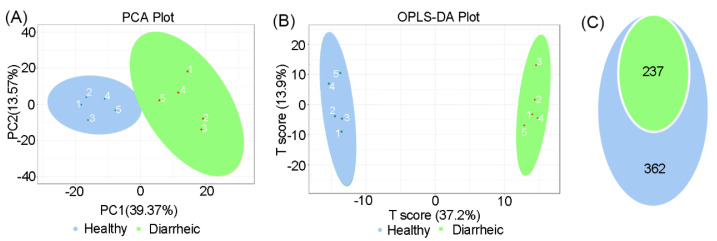
Total metabolite comparisons between groups. PCA score plots (**A**) and OPLS-DA score plots (**B**) based on fecal metabolomics in groups. (**C**) Differential metabolites were identified based on OPLS-DA analysis using *p* < 0.05 and VIP ≥ 1 as filters between groups. Blue = *p* < 0.05; green = *p* < 0.05 and VIP ≥ 1 (*n* = 5 animals/group).

In total, 599 metabolites were detected in all samples. PCA scatter plots showed distinct clustering in fecal metabolite profiles between groups (Figure 3A). An OPLS-DA score chart also showed that groups were clearly separated from each other (R^2^Y = 0.998, Q^2^ = 0.911, Figure 3B). Among metabolites, 237 differential metabolites were identified in the diarrheic group (VIP ≥ 1, *p* < 0.05, Figure 3C). When compared with the healthy group, 120 metabolites were upregulated and 88 metabolites downregulated in the diarrheic group. After filtering using FC ≥ 2 or FC ≤ 0.5 values, 182 metabolites, with greater variability, were further identified (VIP ≥ 1, *p* < 0.05, FC ≥ 2 or FC ≤ 0.5). Therefore, metabolite profile was associated with diarrhea in Père David’s deer.

### 3.4. Altered Metabolites in Diarrheic Père David’s Deer

**Figure 4:**
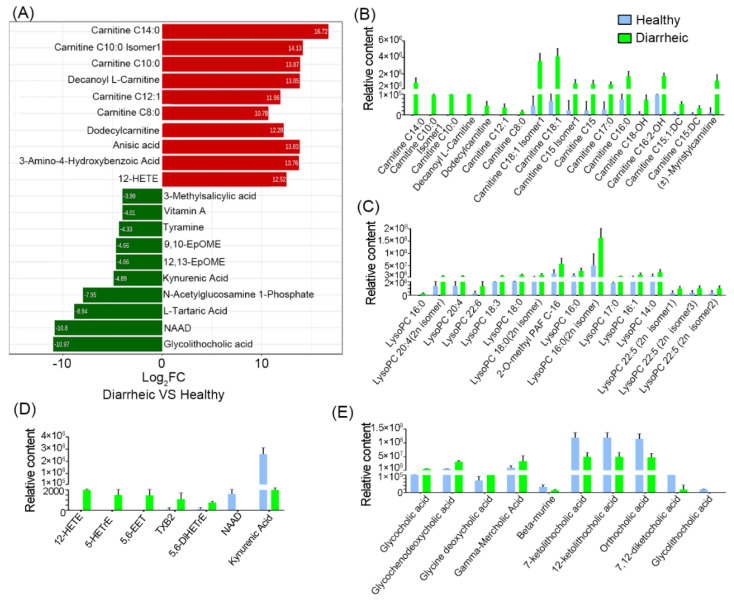
Metabolite comparisons between groups. (**A**) Log_2_ fold change of the top 10 metabolites between groups. Acylcarnitine (**B**), lysophospholipid (**C**), oxidized lipid, NAD, and kynurenic acid (**D**), and bile acid comparisons (**E**). Blue = healthy group; green = diarrheic group. (*n* = 5 animals/group).

Log_2_FC values were calculated, and the top ten increased or decreased metabolites in the diarrheic group, when compared with the healthy group, were identified (Figure 4A). When compared with the healthy group, carnitine (C8:0, C10:0, C14:0, C12:1), carnitine C10:0 Isomer1, decanoyl L-carnitine, dodecylcarnitine, anisic acid, 3-amino-4-hydroxybenzoic acid, and 12-HETE levels were increased > 1000-fold in diarrheic animals; however, vitamin A, tyramine, 9, 10-EpOME, 12,13-EpOME, kynurenic acid, N-acetylglucosamine 1-phosphate, L-tartaric acid, NAD, and glycolithocholic acid levels were decreased by > 16-fold. 

Further analyses showed that 18 acylcarnitines (Figure 4B), 16 lysophospholipids (Figure 4C), and 5 oxidized lipids (Figure 4D) were significantly higher in diarrheic animals. Among these, most acylcarnitines and all oxidized lipids were absent from healthy animal feces, but were dramatically increased in diarrheic animals. Additionally, 10 bile acids were also significantly altered, including several primary bile acids (glycocholic acid and glycochenodeoxycholic acid) and a secondary bile acid (glycine deoxycholic acid), which were significantly higher in diarrheic animals (Figure 4E, *p* < 0.001). However, three secondary bile acid intermediates (7-ketolithocholic acid, 12-ketolithocholic acid, and 7,12-diketocholic acid) and glycolithocholic acid, were significantly lower in the diarrheic group (*p* < 0.001). Moreover, NAD, vitamin A, 4-pyridoxic acid, cyclic AMP, kynurenic acid, and serotonin levels were lower in diarrheic animals. In particular, NAD, vitamin A, and kynurenic acid were almost undetectable in diarrheic feces. Therefore, numerous metabolite changes were identified in diarrhea from Père David’s deer. Especially, some lipid-related metabolites were not present in healthy feces, but were present in large quantities in diarrheic individuals.

### 3.5. Altered Metabolic Pathways in Diarrheic Père David’s Deer

**Figure 5:**
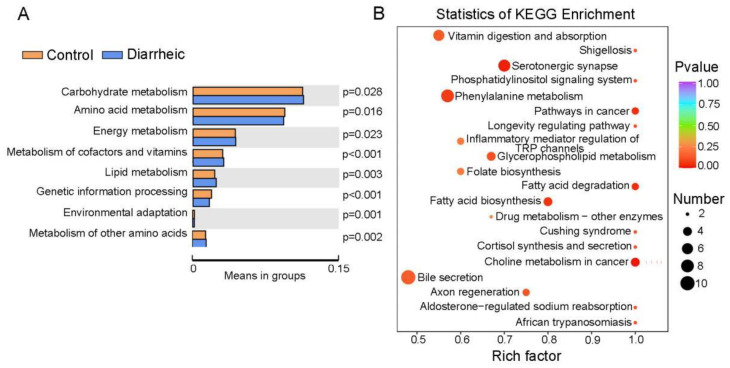
KEGG pathway enrichment analyses of the metabolome and differential metabolites. (**A**) *T*-tests of fecal microbiome pathway-related gene enrichment. (**B**) KEGG enrichment analysis based on 237 differential metabolites (VIP ≥ 1, *p* < 0.05) between healthy group and diarrheic group. The color of the point is the *p*-value; the redder, the more significant the enrichment. Dot size represents the number of enriched differential metabolites.

KEGG analyses revealed a higher abundance of genes associated with carbohydrate, lipid, and energy metabolism in the fecal microbiome of diarrheic animals, while a lower abundance of genes was identified for amino acid metabolism, genetic information processing, and environmental adaptation (Figure 5A). KEGG analyses, based on 237 different metabolites (VIP ≥ 1, *p* < 0.05), showed that more differential metabolites were enriched in bile secretion, fatty acid biosynthesis and degradation, vitamin digestion and absorption, glycerophospholipid metabolism, phenylalanine metabolism, choline metabolism, serotonergic synapse, and inflammatory mediator regulation of Transient Receptor Potential (TRP) channels (Figure 5B), therefore, several pathways were significantly altered in diarrheic animals. Moreover, both bacterial metabolic function estimations and differential metabolites analyses revealed that lipid metabolism, cofactor and vitamin metabolism were altered in diarrheic animals, and suggested the gut microbiota may be involved in these pathways.

### 3.6. Functional Correlations between Fecal Metabolites and Main Gut Microbiota

**Figure 6:**
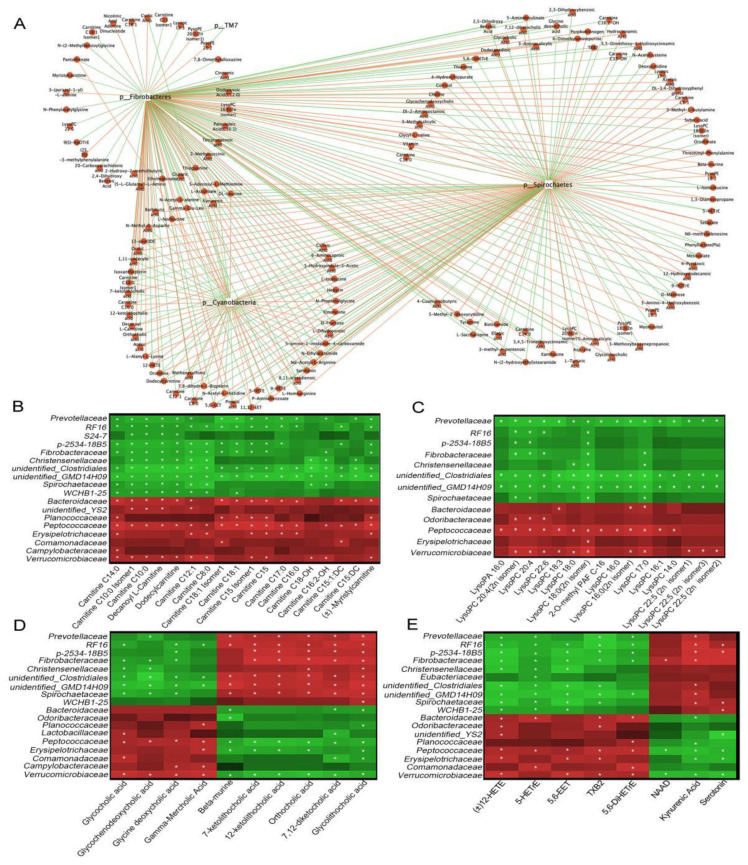
Functional correlations between fecal metabolites and the main microbiota. (**A**) Network diagram showing microbiota at the phylum level, with 182 differential metabolites (VIP ≥ 1, *p* < 0.05, FC ≥ 2 or FC ≤ 0.5). The red line represents a positive correlation, and the green line, a negative correlation. Heat maps represent Spearman correlations for microbiota and differential metabolites of acylcarnitines (**B**), lysophospholipids (**C**), bile acids (**D**), and oxidized lipids (**E**). R values are represented by gradient colors; red and green cells = positive and negative correlations, respectively. Asterisks indicate significance at * *p* < 0.05.

Correlation analyses of different microbes and 182 metabolites (VIP ≥ 1, *p* < 0.05, FC ≥ 2 or FC ≤ 0.5 values), displayed in a network diagram, showed that three phyla (Cyanobacteria, Spirochaetes, and Fibrobacteres) were correlated with most of metabolites (Figure 6A). 52 selected metabolites, such as acylcarnitines, lysophospholipids, and bile acids, displayed positive or negative correlations with the three aforementioned bacterial phyla (Figure S1). Further analyses at the family level was conducted for the 52 selected metabolites. Peptococcaceae and Verrucomicrobiaceae were significantly positively correlated with most acylcarnitines (Figure 6B, *p* < 0.05), lysophospholipids (Figure 6C, *p* < 0.05), several primary bile acids (Figure 6D, *p* < 0.05), and oxidized lipids (Figure 6E, *p* < 0.05), while negatively correlated with secondary bile acids, NAD, and kynurenic acid. Prevotellaceae, unidentified Clostridiales, and unidentified GMD14H09 were significantly negatively correlated with most acylcarnitines, lysophospholipids, and oxidized lipids, while positively correlated with secondary bile acids and kynurenic acid (*p* < 0.05). Additionally, Spirochaetaceae and Bacteroidaceae were significantly correlated with several acylcarnitines, bile acids, oxidized lipids, and kynurenic acid (*p* < 0.05). Thus, the altered metabolites may have partly resulted from the altered gut microbiota in diarrheic Père David’s deer.

### 3.7. Bacteroides Abundance Comparison in Feces of Père David’s Deer with or without Diarrhea

**Figure 7:**
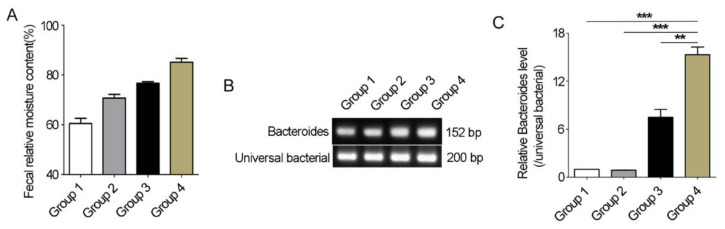
*Bacteroides* abundance comparison in feces of Père David’s deer with or without diarrhea. (**A**) 15 feces samples from non-diarrhea individuals were collected from semi-wide and wild areas and divided into 3 groups (*n* = 5/group) based on relative moisture content. Group 4 was diarrhea feces samples (*n* = 5). (**B**) Conventional PCR was used to amplify *Bacteroides*-specific fragment. (**C**) qRT-PCR was used to compare the *Bacteroides* abundance in Père David’s deer feces with or without diarrhea. Data were presented as the mean ± SEM. ** *p* < 0.01, *** *p* < 0.001.

Using different sources of feces samples, we further detected whether the differences in *Bacteroides* abundance between healthy and diarrheic Père David’s deer really existed. The fecal relative moisture content of fresh feces samples from healthy individuals varied from 40% to 80% (Figure 7A). Conventional PCR with *Bacteroides*-specific primers showed that there were differences between diarrhea and non-diarrhea feces in *Bacteroides* 16S rRNA gene abundance resulting from stronger bands in the diarrhea samples (Figure 7B). qRT-PCR further showed a significant increase in the abundance of *Bacteroides* 16S rRNA gene in diarrheic feces when compared with other feces (*p* < 0.01, Figure 7C). Therefore, the significant differences in *Bacteroides* abundance between healthy and diarrheic Père David’s deer really existed. 

## 4. Discussion

Ruminant intestines are colonized by trillions of microbes that are implicated in immune system maturation, intestinal epithelial mucosal barrier and gastrointestinal (GI) tract maintenance, metabolism, nutrient absorption, and the translocation of intestinal pathogens [31]. The gut microbiota influences essential functions, including digestion, energy metabolism, and inflammation, by modulating multiple host pathways. In our study, a significant reduction in gut microbial abundance and diversity was observed in diarrheic Père David’s deer (Figure 1). Similar data were reported in diarrheic humans (*Homo sapiens*) [32], mice (*Mus musculus*) [33], Baer’s pochards (*Aythya baeri*) [23], musk deer (*Moschus berezovskii*) [19], and giraffes (*Giraffa camelopardalis reticulata*) [24]. Thus, gut microbiota diversity reductions may be typical features in diarrheic individuals, no matter the diarrhea type or causative species. The gut microbiota is a key factor that modulates the host’s energy balance via digested food and produces metabolites and microbial products, such as short-chain fatty acids and secondary bile acids [34]. In turn, these signaling molecules modulate appetite, gut motility, energy uptake and storage, and energy expenditure. Thus, gut microbial abundance and diversity are positively related to intestinal function, and higher levels are conducive to increased energy utilization and complicated physiological functions [35,36]. As with most clinical symptoms of diarrhea, we also observed that the diarrheic deer lose their appetite and are listless and weak. 

A healthy intestinal barrier is characterized by the selective permeability of nutrients, metabolites, water, and bacterial products, while processes are governed by cellular, neural, immune, and hormonal factors [37]. The gut microbiota of Père David’s deer was mostly composed of Firmicutes and Bacteroidetes, at >90% of the total community [2]. We showed that the Firmicutes to Bacteroidetes ratio (2.73) decreased in diarrheic animals when compared with healthy animals (3.47), but this was not statistically significant. Usually, a high Firmicutes/Bacteroidetes ratio maintains a good host metabolic balance via energy harvesting mediated by the gut microbiota [38]. A lower Firmicutes/Bacteroidetes ratio was associated with several pathological conditions in human [39,40]. Our further analysis showed that decreased Firmicutes/Bacteroidetes ratio was mainly due to the significantly decreased abundance of Firmicutes in diarrheic animals (Figure 2). In the Firmicutes phylum, Ruminococcaceae are found in colonic mucosal biofilms in healthy individuals and are regarded as potentially beneficial bacteria as they positively regulate intestinal environments and are linked to immunomodulation and healthy homeostasis [41,42]. Here, significantly decreased Ruminococcaceae abundance was observed in diarrheic Père David’s deer, which is consistent with reports in other types of diarrheas, such as IBS, *Clostridium difficile* infection, *C. difficile*-negative nosocomial diarrhea, and antibiotic-associated diarrhea [43,44]. *Bacteroides* belong to mucus-degrading bacteria, and *Bacteroides* spp. are generally ‘friendly’ commensals in the gut and provide several health benefits to the host [45]. Nevertheless, some *Bacteroides* spp. may have pathogenic roles with regard to intestinal dysfunction, such as diarrhea [46]. Here, *Bacteroides* abundance was significantly higher in diarrheic deer, which was consistent with several reports, such as sunitinib-induced diarrhea in humans, diarrhea in Sichuan golden snub-nosed monkeys (*Rhinopithecus roxellana*), chronic diarrhea in Rhesus macaques (*Macaca mulatta*), and diarrhea in wild boar (*Sus scrofa*) [16,47,48,49]. We hypothesized a relationship between *Bacteroides* abundance and diarrhea that may have been affected by some pathogenic roles from harmful *Bacteroides* spp. That is, an abnormally elevated abundance of *Bacteroides* may be a risk factor for diarrhea. Therefore, we further assessed the *Bacteroides* abundance of the fresh feces with different water contents in the wild and semi-wild areas in Dafeng Reserve using qPCR technology (Figure 7) and made sure that significant differences did exist between diarrheic and non-diarrheic deer. In particular, the *Bacteroides* abundance in diarrheal feces was significantly higher than that in the thinner feces (group 3) that was often observed in the wild area in Dafeng Reserve, indicating the potential of *Bacteroides* abundance as an initial diagnostic marker for the diarrheal diseases for wild and semi-wild areas in Dafeng Reserve. Further study is needed in the future. 

Accumulating evidence has now shown that metabolites are the vital interface between the gut microbiome and host health status [50]. Here, we revealed that gut physiology was distinctly different in diarrheic Père David’s deer (Figure 3). Many fecal metabolites were significantly altered in diarrheic individuals (237 of 599 identified metabolites with VIP ≥ 1, *p* < 0.05 values), which reflected large-scale dysregulation. Bile acids are often analyzed in feces as they are important for host metabolism and they are directly related to intestinal microbiota [51,52]. We observed an excessive loss of fecal primary bile acids (glycocholic and glycochenodeoxycholic acids), and a reduced abundance of secondary bile acid intermediates (7-ketolithocholic acid, 12-ketolithocholic acid, and 7,12-diketocholic acid) in diarrheic feces, indicating bile acid biotransformation by gut bacteria was blocked in diarrheic deer. Indeed, several bacterial families such as Fibrobacteraceae, Spirochaetotaceae, p-2534-18B5, and RF16 were significantly positively correlated with secondary bile acids, and the abundance of these bacteria was significantly decreased in diarrheic deer, which may partly explain the bile acid dysmetabolism by the altered gut microbial ecosystem. 

An essential function of the GI tract is the digestion and turnover of lipids; these molecules constitute a major building material in cell membranes, are a valuable energy source, and are key hormone and signal transduction regulators. Bile acids are essential for the absorption, transport, and metabolism of dietary fats and lipid-soluble vitamins in the intestine [52]. Thus, disordered bile acid metabolism inevitably affects lipid digestion and absorption. Acylcarnitines, lysophosphatidylcholine, and oxidized lipids were abnormally elevated in the feces of diarrheic deer (Figure 4). Lysophospholipids are metabolic intermediates mainly derived from the incomplete hydrolysis of lysoglycerophospholipids and lysosphingolipids. Lysophosphatidylcholine increases pro-inflammatory cytokines and damages the epithelial barrier in IBD [53]. Thus, increased lysophosphatidylcholine levels in the gut may have damaged the epithelial barrier in diarrheic Père David’s deer. 12-HETE is the oxidative metabolite of arachidonic acid and appears to modulate colonic inflammation [54]. In diarrheic Père David’s deer, oxidative metabolites, including 12-HETE, 5-HETrE, and 5,6-EET, were dramatically increased, which may have generated responses to gut injury or stress to modulate inflammatory responses. 

Interestingly, several acylcarnitines (accumulated medium- and long-chain acylcarnitines) were detected in the feces of diarrheic animals. Most acylcarnitines were absent from healthy feces, whereas levels were dramatically increased in diarrheic deer. Several studies examined the relationship between serum acylcarnitines levels and human disease, but little is known about levels in the gut [55,56]. Recently, a fecal study reported that many acylcarnitines were significantly enriched in the feces of dysbiotic IBD; however, their roles remain unknown [28]. Thus, the dramatic increase in medium- and long-chain acylcarnitines may have indicated a severe fatty acid absorption disorder in diarrheic deer. A possible mechanism could be that high levels of fatty acids were released by triglyceride hydrolysis and were then catalyzed by particular gut microbiota to produce acylcarnitines. It is possible that acylcarnitines cannot be absorbed by intestinal epithelial cells and are largely excreted in feces. However, no studies have reported the intestinal microbiota conversion of fatty acids to acylcarnitines. In our study, we observed significant positive correlations between acylcarnitines levels and the abundance of Bacteroidaceae, Peptococcaceae, and Verrucomicrobiaceae, suggesting these families were possibly involved in acylcarnitine transformation (Figure 6). Further studies are required to identify the microbes involved in fatty acid conversion to acylcarnitines in the gut, which may provide new targets to treat diarrhea. In contrast, 9,10-EpOME and 12,13-EpOME, which are linoleic acid metabolites, were significantly lower in diarrheic individuals, and suggested a disturbance in linoleic acid metabolism. Human studies reported that EpOME level was associated with several diseases [57]. 12,13-DiHOME was recently associated with the gut microbiome in young children who developed asthma [58]. Therefore, we hypothesized that decreased EpOMEs may have occurred from decreased GI microbiota responsible for these processes.

Beneficial metabolites such as NAD (active form of niacin), 4-pyridoxic acid (the end product of vitamin B6 catabolism), vitamin A, kynurenic acid, and serotonin were practically undetected in diarrheic deer. Therefore, apart from the effects of lipid metabolism, vitamin synthesis and tryptophan metabolism were also altered in diarrheic deer. A study successfully predicted that 40–65% of human gut microbes have the power to synthesize B vitamins, and riboflavin and niacin were the two most commonly synthesized vitamins by gut microbes [59]. Our KEGG pathway analysis, based on both 16S rRNA gene sequence and differential metabolite analysis, showed that cofactor and vitamin metabolism were altered in diarrheic animals, indicating that the gut microbiota may be involved in cofactor and vitamin alterations in Père David’s deer (Figure 5). Kynurenic acid is the intermediate product of tryptophan metabolism, which critically modulates the gut microbiota and impacts major physiological and pathological pathways [60]. Serotonin is a neurotransmitter required for GI secretion and motility and is associated with the gut microbiota and the gut-brain axis [61]. In mammalian cells, approximately 90% of exogenous tryptophan is converted to kynurenine for further metabolism in the kynurenine pathway, while the remaining tryptophan is metabolized to serotonin and indole [62]. In our study, lower serotonin and kynurenic acid levels suggested decreased tryptophan metabolism in diarrheic deer. 

Diarrhea is a complex disease that can be induced by several infectious and non-infectious causes. Due to insufficient research on diarrhea, veterinarians cannot make an accurate diagnosis of diarrhea that occurs in Dafeng Reserve. So far, there were several potential pathogens identified in Père David’s deer, such *Cryptosporidium*, *Clostridium difficile*, bovine viral diarrhea virus-like strains, and parasites [6,10,63]. However, no evidence has been presented on their effects on the diarrhea in Père David’s deer. Based on our 16S rRNA gene sequencing data, an overabundance of one or more pathogens was not identified in the diarrheic feces. Further study is still urgently needed to identify the content of these reported potential pathogens (parasites, bacteria and viruses) in the feces of diarrheic Père David’s deer. In the current study, the captive areas sampled were about 50 deer. Considering all the diarrheic deer were only 5–8 months old, the diarrhea symptoms occur at the same time, and the diarrhea duration was <15 days, we speculated that the composition of early microbial community gut microbiota in young deer may be more susceptible to diarrheal diseases, regardless of the diarrhea cause. In addition, the limitation of the sick and healthy deer at different ages may lead to differences in the gut microbiota abundance in different age groups. However, we believe that considerable changes of gut microbiome and the profiles of numerous fecal metabolites in diarrheic individuals, are mostly come from the difference between diarrhea and health. Although diarrhea is accompanied by disturbances in microbial composition, our current study further revealed the intestinal ecology changes in diarrheic Père David’s deer, providing important data for guiding treatment of young Père David’s deer with diarrhea. We believed that targeting disordered metabolism of important nutrients may be more effective in preventing and treating diarrhea, together with regulating the gut microbiota using dietary probiotics.

## 5. Conclusions

We revealed the intestinal ecology changes in diarrheic Père David’s deer based on gut microbiome and fecal metabolites analysis. These changes included lower gut microbiota diversity, altered gut microbiota composition, altered fecal metabolite profiles and metabolic pathways. These observations suggested that: (1) intestinal dysbiosis occurred in diarrheic Père David’s deer; (2) several metabolic pathways were seriously disturbed, especially lipid, bile acid, tryptophan, and linoleic acid metabolism; (3) microbiota-host metabolism crosstalk occurred during diarrheal episodes in deer, which caused significant diarrheal symptoms and changes in the intestinal environment. We also found the great changes of several abnormal metabolite levels (acylcarnitines, lysophosphatidylcholine, EpOME and 12-HETEs), which may provide important evaluation markers in the intestinal ecology for diarrhea analyses, then guide the development of scientific nutrient supplementation strategies for sick individuals. In summary, for the first time, we provided important intestinal ecology information for diarrheic Père David’s deer, which may facilitate improved diagnostic and treatment strategies for sick animals in the future.

## Data Availability

All the data that support the findings of this study are available from the corresponding author. Raw sequencing data are deposited into the Sequence Read Archive (SRA; http://www.ncbi.nlm.nih.gov/Traces/sra/, 26 February 2022) of NCBI (SAR: PRJNA809905).

## Supplementary Materials

The following supporting information can be downloaded at: https://www.mdpi.com/article/10.3390/ani12233366/s1, Figure S1: Network diagram of the correlation between 52 differential metabolites and microbiota at the phylum level.
