# Supplementary material for: Intestinal Ecology Changes in Diarrheic Père David’s Deer Revealed by Gut Microbiota and Fecal Metabolites Analysis
Source: Animals (Basel). 2022 Nov 30;12(23):3366. doi: 10.3390/ani12233366 (PMC9737761; doi:10.3390/ani12233366)
Supplement: Supplementary file 1 [file animals-12-03366-s001.zip › animals-1973312-supplementary.pdf]

animals

Supplementary Material

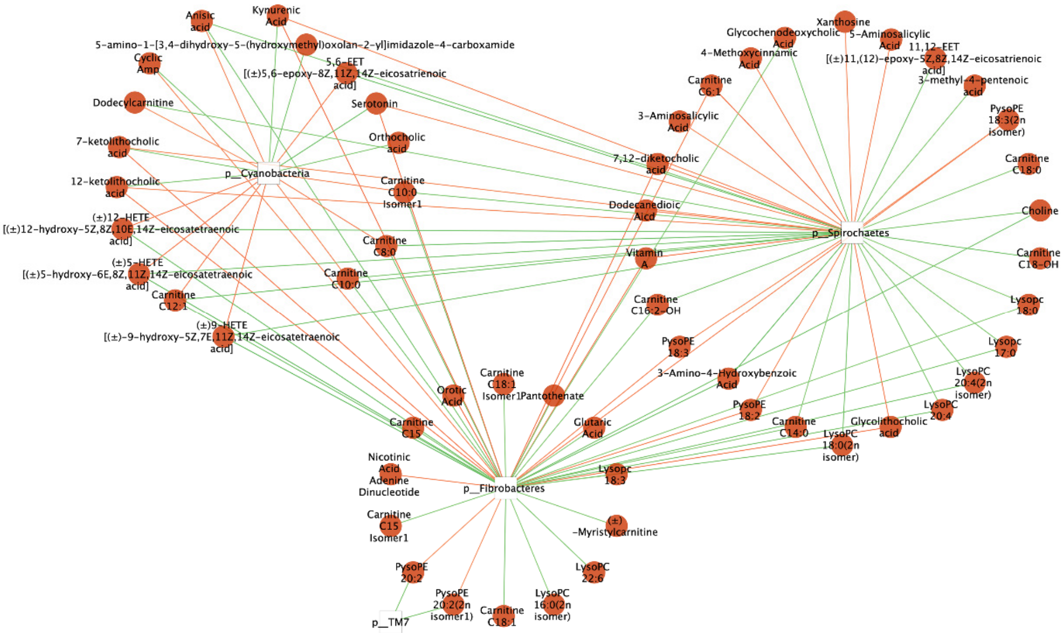

Supplementary Figure S1. Network diagram of the correlation between 52 differential metabolites and microbiota at the phylum level. The red line represents a positive correlation, and the green line, a negative correlation.
